# Aortic valve repair after failed Ross operation in an adolescent

**DOI:** 10.1016/j.xjtc.2025.09.023

**Published:** 2025-11-04

**Authors:** Igor E. Konstantinov, Carolina Rodrigues, Sergei I. Konstantinov, Tyson A. Fricke

**Affiliations:** a Department of Cardiothoracic Surgery, Royal Children's Hospital, Melbourne, Australia; b Department of Paediatrics, University of Melbourne, Melbourne, Australia; c Heart Research Group, Murdoch Children's Research Institute, Melbourne, Australia; d Melbourne Centre for Cardiovascular Genomics and Regenerative Medicine, Melbourne, Australia

**Figure:**
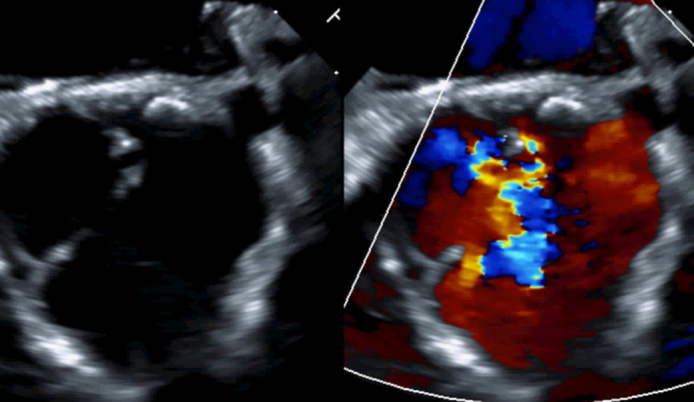
Malcoaptation of all three cusps and wide jet of regurgitation.

**Central Message**

Aortic valve repair after Ross operation should be considered in all patients with a failed autograft.

Aortic valve replacement with a pulmonary autograft provides excellent long-term results.^1^^,^^2^ Nevertheless, failure of an autograft may occur over time. Autograft failure has been found to follow 2 different pathways.^2^ Early presentation is mostly related to primary valve failure as a result of cusp prolapse, whereas late failure is associated with autograft root dilatation^E1^ and tends to develop gradually, with the greatest progression in the first 5 years.E2, E3, E4 The ideal technique of valve-sparing aortic valve repair after failed Ross operation is yet to be determined.

Herein, we describe the surgical management of an adolescent patient with severe aortic valve regurgitation after having a Ross operation at 13 years of age using a root inclusion technique. The Royal Children's Hospital Human Research Ethics Committee approved this retrospective review (HREC/21/QCHQ/80891; November 11, 2021), and the parents of the patient provided informed written consent for the publication of the data.

## Case Report

A 16-year-old boy (weight 70 kg, height 178 cm, body surface area 1.9 m^2^) presented with autograft failure after Ross operation. He initially had unicuspid aortic valve with complete commissural fusion between the left coronary and the right coronary cusps as well as between the noncoronary and the right coronary cusps. At the age of 2 years, he presented with severe stenosis and moderate regurgitation and underwent aortic valve repair with commissurotomy between the non- and right coronary cusps and resection of raphe between the left and the right coronary cusps and trigonal pericardial patch repair at the site of commissurotomy. At 13 years of age, he underwent Ross operation with a 24-mm pulmonary homograft and the use of root inclusion technique. He still had excellent exercise tolerance. Because of his current sport activities, systemic anticoagulation was contraindicated.

**Figure 1:**
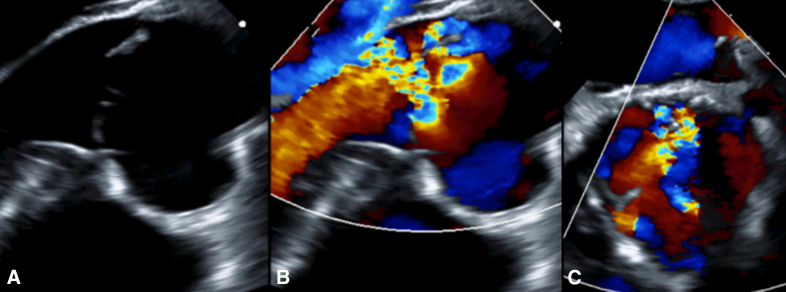
Preoperative echocardiogram demonstrated severe aortic regurgitation in long-axis (A, B) and short-axis views (C).

Preoperative echocardiogram demonstrated dilated left ventricle (LV) with an LV end-diastolic volume of 171 mL/m^2^ (z score 2.3) and LV end-systolic volume of 72 mL/m^2^ (z score 2.1). He had mildly reduced LV systolic function with an LV ejection fraction of 58% and a dilated aortic valve annulus of 30 mm. He also had severe aortic valve regurgitation (Figure 1) with mild stenosis (mean pressure gradient of 15.6 mm Hg), a dilated ascending aorta with aortic sinuses of 3.3 cm (normal <3.2 cm), and a sinotubular junction of 1.9 cm (normal <2.7 cm).

**Figure 2:**
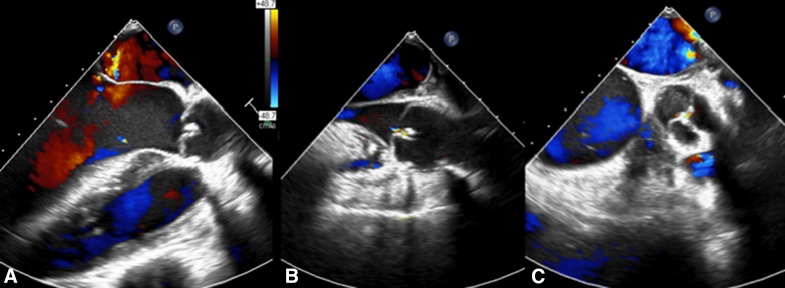
Postoperative echocardiogram demonstrated mild aortic regurgitation and good leaflet coaptation in long-axis view during early diastole (A), late diastole (B) and in short-axis view (C).

The aorta was opened and cardioplegia was delivered separately in each ostium. The aortic valve was examined (Video 1). Aortic valve repair was performed and consisted of 3 steps. First, annular reduction was performed using GoreTex (W. L. Gore & Associates) suture (CV-0) so that the aortic valve annulus was reduced around a 25-mm Hegar dilator. The GoreTex suture was tightened from the outside above the roof of the left atrium and a titanium clip were placed on it to avoid inadvertent loosening of the annuloplasty suture with time. Second, the noncoronary and the right coronary cusps were plicated in the middle of the free edge to create a new nodule of Arantius in each cusp and, thus, to reduce the length of the free edge, increase the effective height and improve the coaptation of the cusps. Third, the sinotubular junction was stabilized with a 24-mm Intergard vascular graft (Intervascular SAS) conduit. The patient was weaned off cardiopulmonary bypass without any inotropes. Aortic crossclamp time was 96 minutes and cardiopulmonary bypass time was 146 minutes. Postoperative echocardiogram demonstrated mild aortic regurgitation and good ventricular function (Figure 2). The patient is doing very well at 8 months of follow-up with same echocardiographic findings.

## Comment

The failed autograft after Ross operation is often replaced with a stented valve prosthesis,^E5^^,^^E6^ with or without concomitant replacement of aortic root,^E5^^,^^E6^ exposing the patients to the potential problems that were meant to be avoided after Ross operation. The development of valve-sparing root replacement and aortic valve repair are alternative approaches that might preserve the advantages of the Ross operation. We only found 4 studies, describing 25 to 30 patients each, in which valve preservation was used in the majority of patients submitted to autograft reoperation.^3^^,^^E1^^,^^E7^^,^^E8^ Surgical options described in these studies include root remodeling, aortic valve reimplantation, correction of sinotubular dilatation, external suture annuloplasty, and central cusp plication.^3^^,^^E1^^,^^E7^^,^^E8^ Not surprisingly, isolated cusp repair has been associated with high rates of recurrent autograft regurgitation.^3^^,^^4^ It now becomes clear that stabilization of the aortic root, at both aortic annular and sinotubular junction levels, is a key factor for long-term durability of any aortic valve repair, including that after failed Ross operation.^5^ In a multicenter study, estimated freedom from reintervention at 8 years of follow-up after a valve-sparing reoperation was 33% in patients with isolated autograft valve repair, compared with 85% in patients in whom aortic root was stabilized, with or without cusp repair.^4^ Therefore, we believe that combined root and cusp repair approach is essential to achieve and maintain a normal valvular function.

## Conflict of Interest Statement

The authors reported no conflicts of interest.

The *Journal* policy requires editors and reviewers to disclose conflicts of interest and to decline handling or reviewing manuscripts for which they may have a conflict of interest. The editors and reviewers of this article have no conflicts of interest.

## Supplementary Data

Video 1 The senior author explains perioperative findings and procedure. Video available at: https://www.jtcvs.org/article/S2666-2507(25)00444-4/fulltext.

## References

[1] Buratto E., Wallace F.R.O., Fricke T.A. (2020). Ross procedures in children with previous aortic valve surgery. J Am Coll Cardiol.

[2] Donald J.S., Wallace F.R.O., Naimo P.S. (2020). Ross operation in children: 23-year experience from a single institution. Ann Thorac Surg.

[3] de Kerchove L., Boodhwani M., Etienne P. (2010). Preservation of the pulmonary autograft after failure of the Ross procedure. Eur J Cardiothorac Surg.

[4] Mookhoek A., de Kerchove L., Khoury E. (2015). European multicenter experience with valve-sparing reoperations after the Ross procedure. J Thorac Cardiovasc Surg.

[5] Schafers H.J., Konstantinov I.E. (2025). Surgical anatomy of aortic root; towards precise and durable aortic, neo-aortic, and truncal valve repair. J Thorac Cardiovasc Surg.

