# Supplementary figures and images for: Aortic valve repair after failed Ross operation in an adolescent
Source: JTCVS Tech. 2025 Nov 4;34:141–4. doi: 10.1016/j.xjtc.2025.09.023 (PMC12683046; doi:10.1016/j.xjtc.2025.09.023)

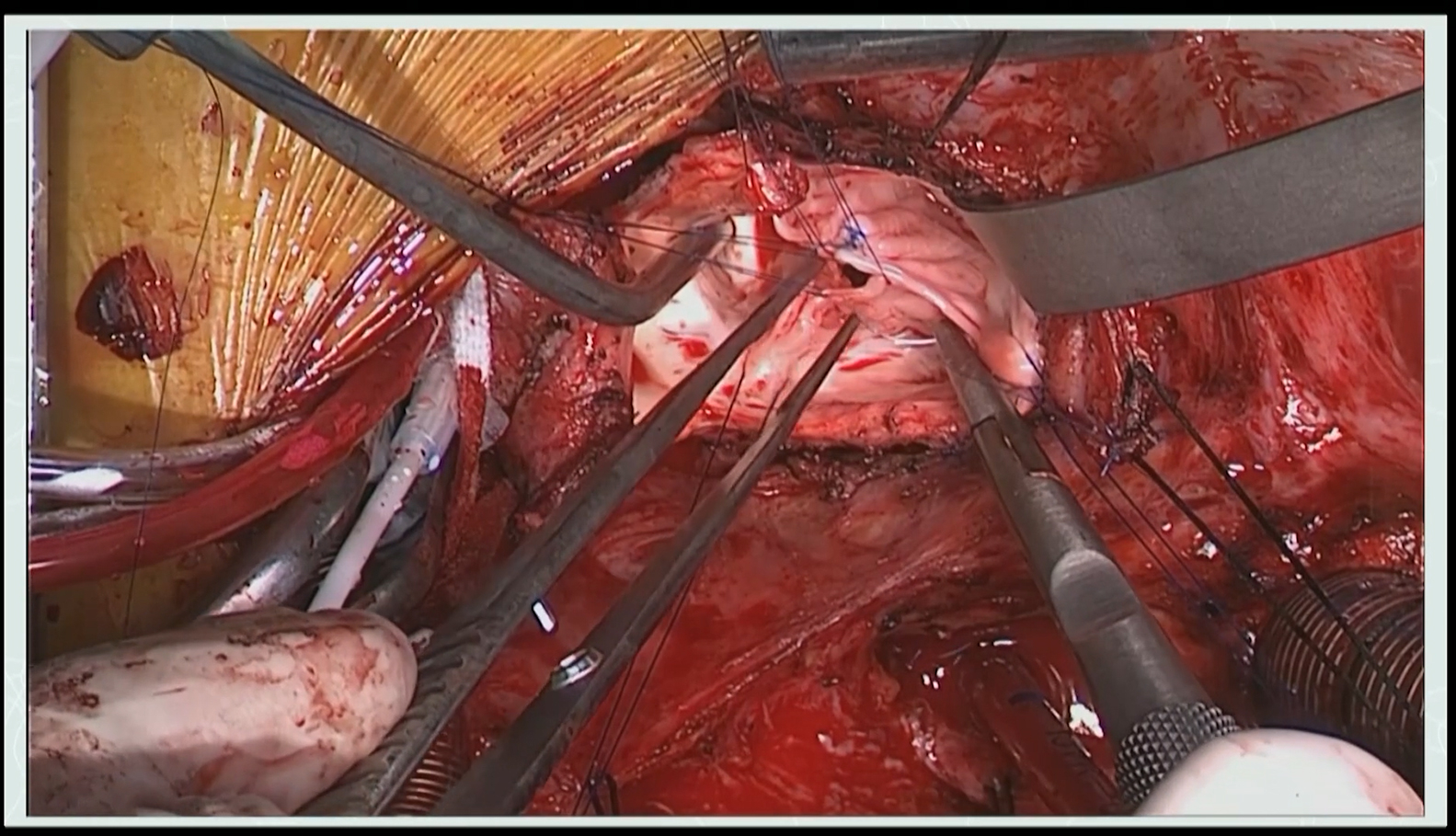

Supplement: Video 1 — The senior author explains perioperative findings and procedure. Video available at: https://www.jtcvs.org/article/S2666-2507(25)00444-4/fulltext. [file fx2.jpg]
